# 48 h Normothermic Machine Perfusion With Urine Recirculation for Discarded Human Kidney Grafts

**DOI:** 10.3389/ti.2023.11804

**Published:** 2023-10-13

**Authors:** Franka Messner, Afschin Soleiman, Dietmar Öfner, Hannes Neuwirt, Stefan Schneeberger, Annemarie Weissenbacher

**Affiliations:** ^1^ Department of Visceral, Transplant and Thoracic Surgery, Center of Operative Medicine, Medical University of Innsbruck, Innsbruck, Austria; ^2^ INNPATH, Institute of Pathology, Tirol Kliniken Innsbruck, Innsbruck, Austria; ^3^ Department of Internal Medicine IV, Nephrology and Hypertension, Medical University of Innsbruck, Innsbruck, Austria

**Keywords:** machine perfusion, kidney preservation, normothermic machine perfusion for the donor kidney, organ assessment, urine recirculation, *ex vivo* perfusion

## Abstract

Normothermic machine perfusion (NMP) has reshaped organ preservation in recent years. In this preclinical study, prolonged normothermic perfusions of discarded human kidney grafts were performed in order to investigate perfusion dynamics and identify potential quality and assessment indicators. Five human discarded kidney grafts were perfused normothermically (37°C) for 48 h using the Kidney Assist device with a red-blood-cell based perfusate with urine recirculation. Perfusion dynamics, perfusate and urine composition as well as injury markers were measured and analyzed. Donor age ranged from 41 to 68 years. All but one kidney were from brain dead donors. Perfusions were performed successfully for 48 h with all discarded kidneys. Median arterial flow ranged from 405 to 841 mL/min. All kidneys excreted urine until the end of perfusion (median 0.43 mL/min at the end of perfusion). While sodium levels were consistently lower in urine compared to perfusate samples, this was only seen for chloride and potassium in kidney KTX 2. Lactate, AST, LDH as well as pro-inflammatory cytokines increased over time, especially in kidneys KTX 3 and 4. *Ex vivo* normothermic perfusion is able to identify patterns of perfusion, biological function, and changes in inflammatory markers in heterogenous discarded kidney grafts.

## Graphical Abstract

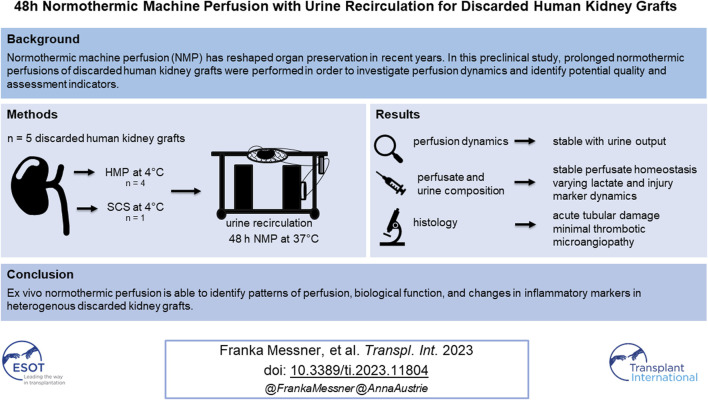

## Introduction

Normothermic machine perfusion (NMP) of donor organs has seen an unprecedented interest in recent years. While liver, lung and heart NMP has become clinical routine, standard implementation of kidney NMP is still lagging behind. In contrast to other organs, kidney grafts tolerate a much longer cold ischemic time even with static storage, thus ease of logistical constrains is a less driving force in this setting. More momentum for innovative approaches is generated by a yearly growing organ shortage and a marked increase in marginal donors.

Thus far, kidney NMP has mainly been explored in marginal, namely extended criteria donors (ECD) and donation after cardiocirculatory death donors (DCD), donors with relatively short perfusion durations [1–5]. In their latest publication, [6] reported on their randomized controlled trial that compared 170 kidneys with 1 h (hr) end-ischemic NMP to 168 statically cold stored (SCS) kidneys. With comparable rates of thrombosis, infectious complications, delayed graft function and other adverse events they demonstrated feasibility and safety for clinical application, however, NMP in this setting did not lead to superior short-term outcomes whilst adding a logistical burden.

In contrast to this end-ischemic approach, prolonged perfusion might be a tool to assess and/or condition organs prior to transplant. Longer perfusion times might also prove to be a logistic advantage by increasing summative preservation times and consequently help to increase organ utilization.

In this preclinical study, discarded human kidney grafts were perfused on the Kidney Assist device with urine recirculation in order to 1) describe perfusion dynamics, 2) investigate biological function, and 3) report on changes in inflammatory markers in a heterogenous group of kidneys over 48 h.

## Materials and Methods

The human kidney grafts included in this study were retrieved for transplant but eventually declined at the recipient center. Experimental perfusions for 48 h were performed in the laboratory of the Organ Regeneration Center of Excellence, organ-Life™, Medical University of Innsbruck after approval by the institutional ethics committee (EK Nr. 1216/2019).

### Perfusion Set-Up

Custodiol® HTK (histidine-tryptophan-ketoglutarate; Dr. Franz Köhler Chemie GmbH, Bensheim, Germany) solution preserved kidneys arrived at the transplant center statically cold stored. Grafts were routinely placed on the LifePort kidney transporter (Organ Recovery Systems, Itasca, IL, USA) immediately after arrival. After deemed untransplantable, the organ was taken off the hypothermic machine perfusion (HMP) device and prepared for connection to the NMP circuit. In one case, no HMP was performed due to logistical reasons and the kidney graft was perfused normothermically after routine back table preparation and static cold storage in Custodiol® HTK.

For NMP, the Organ Assist Kidney Assist (Organ Assist BV, Groningen, Netherlands) device was used. The renal artery was cannulated with a 20-Fr (KTX 1, KTX 2) or 16-Fr (KTX 3, KTX 4, KTX 5) straight perfusion cannula (Infusion, Warsaw, Poland). The ureter was cannulated with the provided tubing. A three-way valve was included in the tubing to allow for urine collection and recirculation into the reservoir. The disposable set was adapted by implementing an in-line blood gas analyzer (CDI500, Terumo Medical Corporation, Tokyo, Japan). Perfusate temperature was set at 37°C. Oxygenation of the circuit was facilitated by manual regulation of air (21% oxygen) and CO_2_. The perfusion circuit was primed with three units of packed red blood cells (RBCs) of universal donor blood, resuspended in 1000 mL 5% human albumin solution (Albunorm®, Octapharma, Lachen, Switzerland), resulting in a total perfusate volume of approximately 1800 mL. The protocol was adapted from the protocol published by Weissenbacher et al. [7] Before connecting the kidney, the perfusate was supplemented with 750 mg cefuroxime (Sandoz, Basel, Switzerland), 10 mL calcium gluconate 10% (B.Braun, Melsungen, Germany), and 8000 IE enoxaparin (Lovenox®, Sanofi, Paris, France). For pH adjustment prior to initiation of NMP, 10 mL of sodium bicarbonate 8.4% (Fresenius Kabi, Bad Homburg, Germany) were added to the perfusate to achieve a pH level >7.0. Immediately after perfusion start, 5 mL verapamil (Mylan, Vienna, Austria) was administered directly into the arterial line. Kidneys were perfused with a median arterial pressure (MAP) of 90 mmHg. MAP was lowered to 80 mmHg in case flow reached 900 mL/min due to flow restrictions of the device (KTX 1 at 40 h and KTX 2 at 24 h into the perfusion). For glucose and electrolyte monitoring, blood gas analyzer (BGA) measurements (ABL800Flex, Drott Medizintechnik GmbH, Wiener Neudorf, Austria) were performed throughout the perfusion and total parenteral nutrition (Nutriflex® plus, containing 0.15 g/mL glucose) was administered once perfusate glucose levels dropped below 70 mg/dL. Data from the first perfused kidney have already been published as a proof of principle study [8]. To give a better overview on perfusion dynamics and explore the potential of kidney NMP in graft assessment, we included this kidney in this manuscript.

### Sampling Procedure

Perfusate and urine samples were obtained throughout the duration of perfusion at hrs 1, 6, 12, 20, 24, 40, and 48. Samples were analyzed upfront by blood gas analyzer and institutional biochemistry laboratory as well as stored after centrifugation at 15,000 G for 15 min at −80°C.

In all kidney grafts, a zero-biopsy was performed and the Remuzzi score was assessed by the on-call pathologist. Follow-up biopsies were taken after 24 h of NMP and upon reaching the endpoint at 48 h. Hemodynamic perfusion parameters were recorded at corresponding timepoints.

### Luminex

Kidney injury markers (Luminex Performance Human Kidney Biomarker Panel [6-Plex], #FCSTM16-06, R&D Systems, Minneapolis, United States) and cytokine (Luminex Performance Human High Sensitivity Cytokine Panel A [12-Plex], #FCSTM09-12, R&D Systems, Minneapolis, United States) levels were measured in perfusate samples stored at −80°C. Sample dilution, processing and analysis were carried out according to manufacturer’s instruction.

### Statistical Analysis

Results are expressed as median and interquartile range (IQR) or range. Mann-Whitney test, Kruskal Wallis, Friedman test corrected with Dunn’s multiple comparison test and Wilcoxon matched-pairs signed rank test were used for non-normal distributed data. All tests were two-sided and a *p*-value of <0.05 was considered statistically significant. Prism GraphPad 9.0 (GraphPad Inc., San Diego, CA, USA) was used for all statistical tests.

## Results

### Donor and Kidney Graft Demographics

Donor age ranged from 41 to 68 (median 62) years (yrs). Four out of five kidney grafts were from DBD donors, only one (KTX 3) was from a DCD donor. Causes of death were all cardiovascular events. All but one donor had a normal ranged serum creatinine level (median 1.06 mg/dL) before organ retrieval. One donor (KTX 4) was on veno-venous extracorporeal membrane oxygenation (vv-ECMO) and renal replacememt therapy (RRT) due to severe aspiration and concomitant sepsis. All donors had urine output before organ retrieval (median 85 mL/h). Two donors had a history of hypertension, none of the donors were diabetic. For details see Table 1.

**TABLE 1:** Donor demographics.

	**KTX 1**	**KTX 2**	**KTX 3**	**KTX 4**	**KTX 5**
Donor age (years)	62	68	51	41	65
Donor type	DBD	DBD	DCD	DBD	DBD
Cause of death	CVA	CVA	CVA	CVA	CVA
Creatinine (mg/dL)	0.9	1.06	1.17	1.98 (RRT)	0.74
Urine production (mL/h)	100	85	117	52	70
Hypertension	unknown	Yes	no	no	yes
Diabetes	no	No	no	no	no
Comment	suspected malignancy			VV-ECMO due severe to aspiration; sepsis	

DBD, donation after brain death; DCD, donation after cardiocirculatory death; CVA, cardiovascular accident; RRT, renal replacement therapy.

Cold ischemic times ranged from 11.5 to 28 (median 19.5) hrs. All but one kidney were perfused hypothermically after being transported to our unit on static cold storage (SCS) using the Lifeport® device for a median of 7 h. Reasons for discard were malignancy in the contralateral kidney, poor organ quality and poor perfusion. Remuzzi scores ranged between 0 and 5 (median 3). A detailed overview can be found in Table 2.

**TABLE 2:** Kidney baseline parameters.

	**KTX 1**	**KTX 2**	**KTX 3**	**KTX 4**	**KTX 5**
CIT total (hours)	27	19.5	19	28	11.5
HMP duration (hours)	4.5	5	10	9.5	n.a.
Reason for discard	malignancy	poor organ quality^a^	poor perfusion	poor perfusion	poor organ quality^b^
Remuzzi score	0	4	3	2	5

CIT, cold ischemic time; HMP, hypothermic machine perfusion.

^a^ large cyst and Remuzzi score: g1, i1, t1, a1 = 4.

^b^ Remuzzi score: g1, i1, t1, a2 = 5.

### Perfusion Characteristics

**FIGURE 1:**
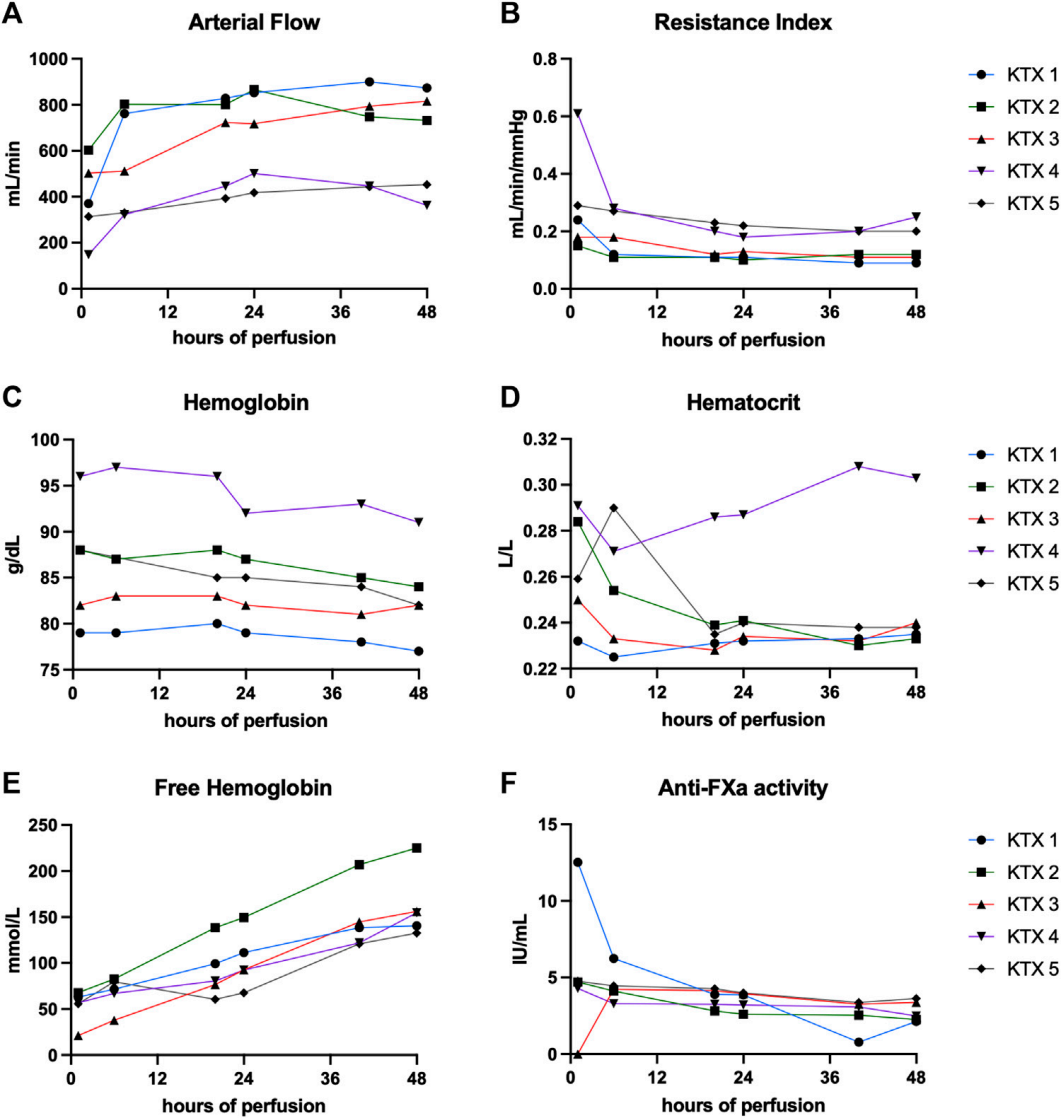
Perfusion characteristics and composition over 48 h of perfusion. **(A)** Arterial flow, corresponding resistance indices **(B)**, hemoglobin levels **(C)** and hematocrit **(D)**, free hemoglobin **(E)** and anti-FXa activity **(F)** is shown at hours 1, 6, 20, 24, 40, and 48.

Arterial flow was stable throughout the perfusion of all kidney grafts (Figure 1A) irrespective of reason for discard. In the first three perfused kidneys, the median arterial flow exhibited higher values, measuring 841, 775, and 721 mL/min, in contrast to the second pair of kidneys, which recorded flow rates of 405 and 406 mL/min, respectively (*p* < 0.001). Correspondingly, resistance indices (Figure 1B) were lower in the first three kidneys compared to the last two (*p* < 0.001). Median hemoglobin and hematocrit levels ranged between 9.5 and 7.9 g/dL, and 0.23 and 0.29 L/L (Table 3; Figures 1C, D). Free hemoglobin increased over time (Figure 1E). Median free hemoglobin levels ranged from 74 to 144 mg/dL (Table 3). Anti-FXa activity ranged between 2.7 and 4.1 UI/mL. Levels were slowly decreasing over time and only KTX2 demonstrated a clear clearance during perfusion (Figure 1F).

**TABLE 3:** Perfusion characteristics. Additive use (total amount), perfusion characteristics (median [IQR]) as well as biochemical markers (median [IQR]) throughout the 48 h perfusion.

	**KTX 1**	**KTX 2**	**KTX 3**	**KTX 4**	**KTX 5**	***p*-value**
Sodium bicarbonate (mL)	15	32	32	40	20	
Nutriflex (mL)	55	45	69	65	35	
Arterial flow (mL/min)	841 (664–881)	775 (700–819)	721 (510–800)	405 (279–461)	406 (326–446)	<0.001
Resistance index (mmHg/mL/min)	0.11 (0.15–0.09)	0.12 (0.13–0.11)	0.13 (0.18–0.11)	0.23 (0.36–0.20)	0.23 (0.28–0.20)	<0.001
pH	7.15 (7.08–7.15)	7.29 (7.02–7.37)	7.16 (7.08–7.22)	7.09 (6.87–7.20)	7.34 (7.22–7.40)	0.070
Lactate (mg/dL)	89.5 (86–102)	119 (81–136)	114 (93.3–194)	179 (143–183)	98.5 (86.5–112)	0.063
Venous pO_2_ (mmHg)	113 (125–107)	79.9 (86.9–78.5)	81.9 (107–76.2)	90.3 (97.4–79.7)	80.3 (89.2–73.8)	<0.001
Venous pCO_2_ (mmHg)	37.8 (39.6–36.1)	34.6 (37.8–31.2)	36.3 (40.7–29.1)	13.4 (22.2–10.8)	24.8 (32.5–19.8)	<0.001
Sodium (mmol/L)	158 (159–156)	163 (163–153)	167 (169–163)	173 (177–158)	176 (177–171)	0.001
Chloride (mmol/L)	128 (129–121)	118 (122–110)	119 (123–117)	117 (117–114)	135 (136–131)	<0.001
Potassium (mmol/L)	6.85 (8.03–6.48)	9.95 (11.2–9.23)	6.00 (7.08–5.83)	8.15 (9.68–6.7)	6.35 (7.23–6.15)	<0.001
Hemoglobin (g/dL)	7.9 (7.93–7.78)	8.7 (8.8–8.48)	8.2 (8.3–8.18)	9.45 (9.63–9.18)	8.5 (9.2–8.35)	<0.001
Hematocrit (L/L)	0.23 (0.23–0.23)	0.24 (0.26–0.23)	0.23 (0.24–0.23)	0.29 (0.30–0.28)	0.24 (0.27–0.24)	0.005
Free hemoglobin (mg/dL)	10 (139–69.4)	144 (212–78.8)	84.5 (148–33.7)	86.5 (130–64.5)	73.6 (124–59.3)	0.004
Anti-FXa activity (IU/mL)	3.9 (7.8–1.8)	2.7 (4.3–2.5)	3.7 (4.2–2.4)	3.2 (3.5–2.9)	4.1 (4.5–4.6)	0.018
Urine (mL/min) at hour 48	0.43	0.56	0.37	1.14	0.40	
Urine sodium (mmol/L)	138 (151–109)	117 (137–100)	163 (163–144)	163 (174–121)	100 (171–71)	0.009
Urine choride (mmol/L)	123 (131–109)	102 (119–80)	123 (124–120)	124 (126–105)	98 (140–87)	0.063
Urine potassium (mmol/L)	7.65 (9.88–6.83)	16.2 (17.7–15.9)	6.7 (11.1–5.79)	9.45 (20.8–8.43)	15.8 (19.1–6.50)	0.009
Proteinuria, absolute (mg/dL)	3,964 (6,072–1,856)	9,310 (22,983–5,177)	30,825 (32,516–28,852)	28,251 (28,891–19,273)	6165 (34,468–521)	0.029

GOT, Glutamic-Oxaloacetic Transaminase; IQR, interquartile range; LDH, Lactate Dehydrogenase; pO_2_, partial pressure of oxygen; pCO_2_, partial pressure of carbon.

Median (IQR) venous pO_2_ und pCO_2_ levels were 113 (125–107) mmHg and 37.8 (39.6–36.1) mmHg for KTX 1, 79.9 (86.9–78.5) mmHg and 34.6 (37.8–31.2) mmHg for KTX 2, 81.9 (107–76.2) mmHg and 36.3 (40.7–29.1) mmHg for KTX 3, 90.3 (97.4–79.7) mmHg and 13.4 (22.2–10.8) mmHg for KTX 4, and 80.3 (89.2–73.8) mmHg and 24.8 (32.5–19.8) mmHg for KTX 5.

**FIGURE 2:**
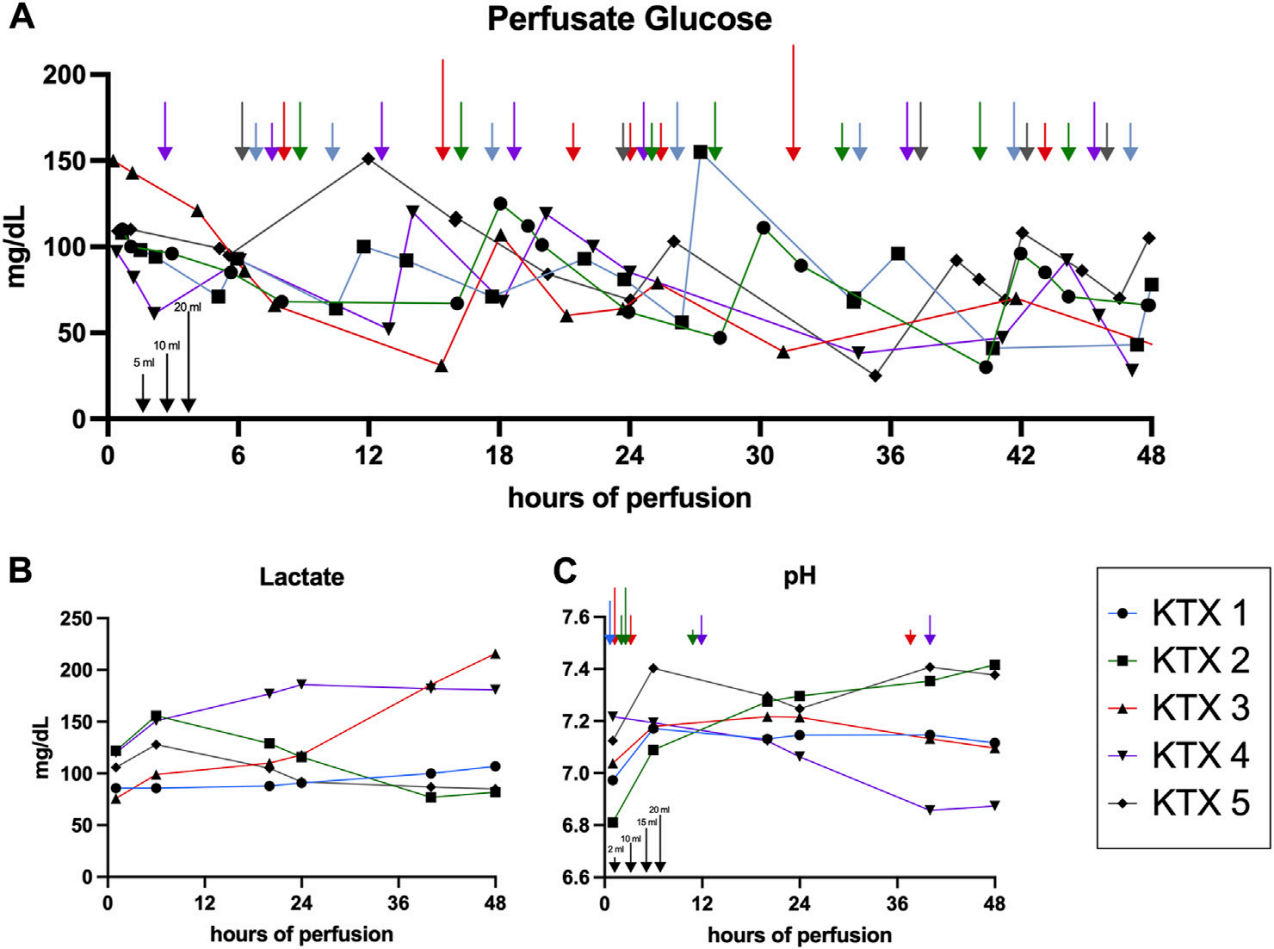
Glucose, lactate and pH dynamics over 48 h of perfusion. **(A)** Glucose concentration assessed via perfusate blood gas analysis and resulting Nutriflex supplementation over time. Arrows indicate the Nutriflex amount administered at a given timepoint (blue, KTX 1; green KTX 2; red, KTX 3; purple, KTX 4; grey, KTX 5; for amount see indicators at the bottom left). **(B)** Lactate dynamics and **(C)** pH fluctuation and supplementation of sodium bicarbonate (blue, KTX 1; green KTX 2; red, KTX 3; purple, KTX 4; grey, KTX 5; for amount see indicators at the bottom left) for buffering over time.

**FIGURE 3:**
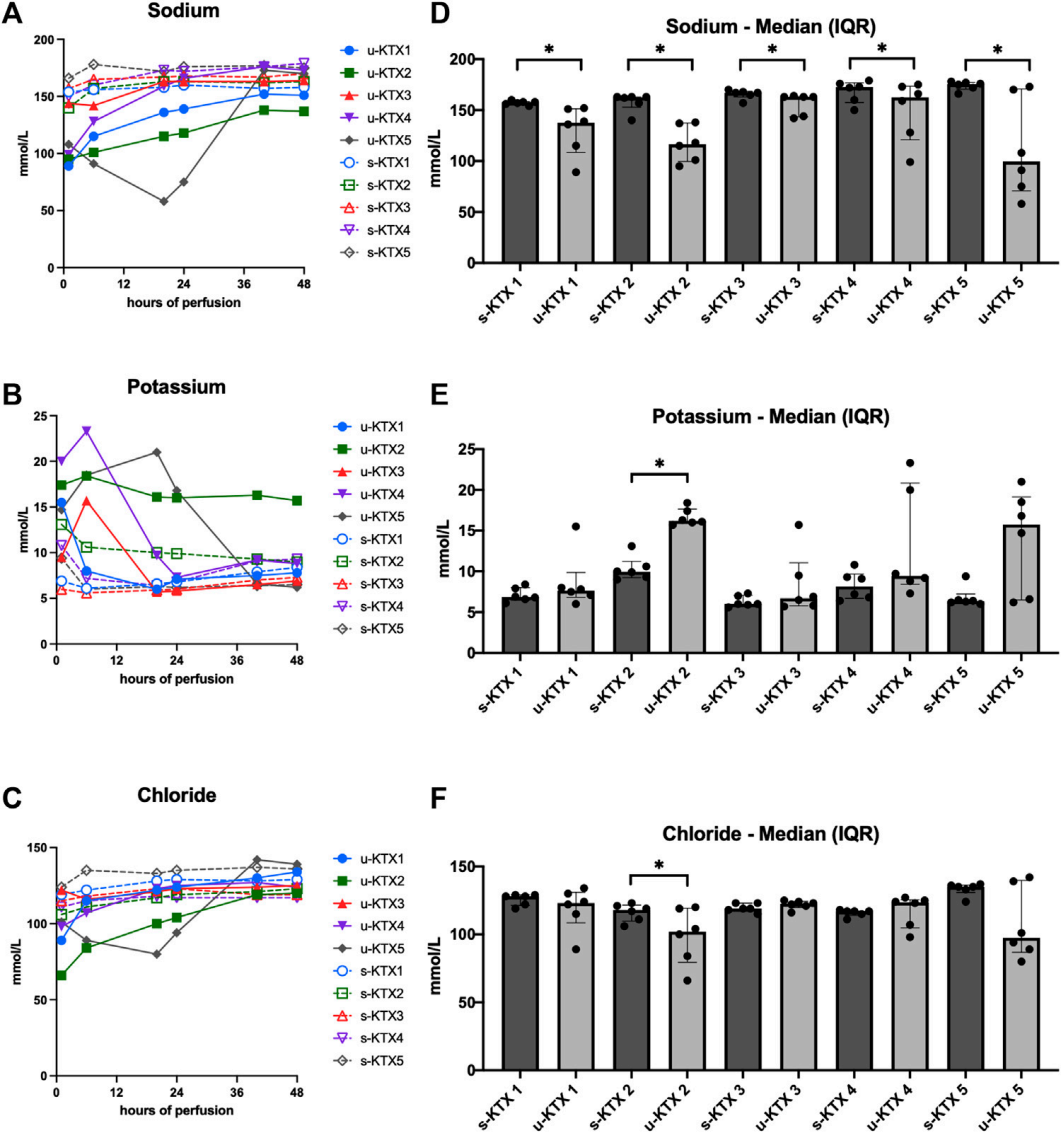
Perfusate and urine electrolyte levels. **(A–C)** Absolute sodium, potassium and chloride concentrations in serum (s-KTX, solid line) and urine (u-KTx, interrupted line). **(D–F)** Median (IQR) perfusate (s-KTX) and urine electrolyte levels (u-KTX) over the course of the 48 h perfusion were illustrated and compared using the Wilcoxon matched-pairs signed rank test. *p < 0.05; IQR, interquartile range.

With 35–69 mL of summative Nutriflex supplementation, glucose concentrations between 50 and 150 mg/dL were achieved (Figure 2A). Lactate levels ranged between 179 and 89.5 mg/dL. Even tough median lactate levels were similar between groups (*p* = 0.063), lactate dynamics differed. While lactate was cleared in KTX 1 and 5, it remained stable in KTX 2 and increased over the duration of perfusion in KTX 3 and 4 (Figure 2B). Supplementation of 15–40 mL sodium bicarbonate were necessary to achieve median pH levels between 7.09 and 7.34 (Table 3; Figure 2C). Despite a uniform basic perfusate formulation, kidneys exhibited different electrolyte levels in the perfusate (Table 3; Figure 3). Perfusate sodium and chloride levels increased over the first 24 h of perfusion and stabilized afterwards (Figures 3A, C). Perfusate potassium levels, on the contrast, decreased over the course of the first 24 h in all cases. Thereafter it further decreased, stabilized or increased depending on potassium loss via urine sampling (Figure 3B).

All kidney grafts had urine output throughout the perfusion. Median urine output was 0.43 mL/min at the end of perfusion. Median urine sodium content was significantly lower than in the perfusate in all perfused kidney grafts (Figure 3D, *p* = 0.031). In contrast, median chloride (Figure 3F, *p* = 0.031) and potassium (Figure 3E, *p* < 0.001) levels differed only in KTX 2.

### Injury Markers

**FIGURE 4:**
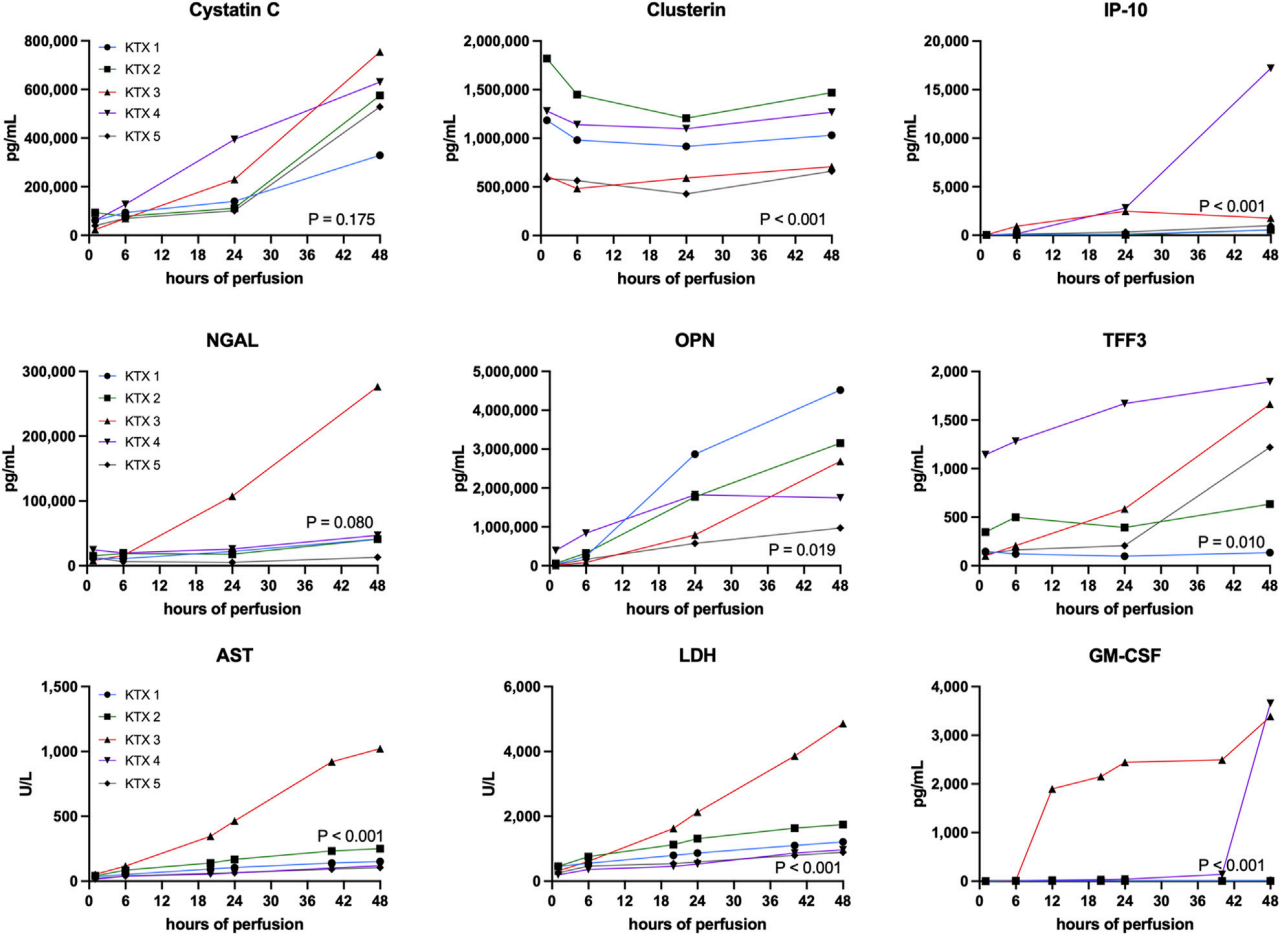
Quantification of injury markers. Unspecific and kidney specific injury markers have been measured at corresponding timepoints throughout the 48 h perfusion. A comparison between perfused kidneys was performed using the Friedman test. AST, aspartate aminotransferase; GM-CSF, granulocyte macrophage colony-stimulating factor; LDH, lactate dehydrogenase; IP-10, CXCL10; NGAL, Neutrophil Gelatinase-associated Lipocalin; OPN, Osteopontin; TFF3, Trefoil Factor 3.

**FIGURE 5:**
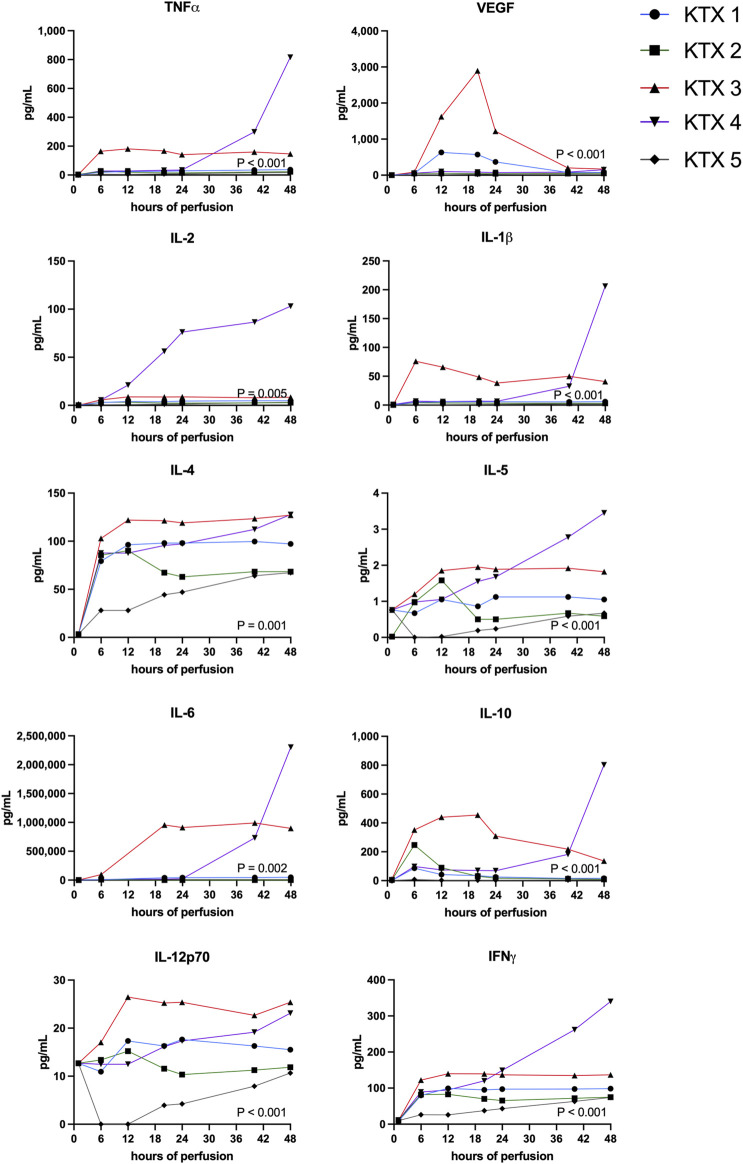
Pro- and anti-inflammatory protein perfusate levels during 48 h kidney NMP were measured and compared between perfused kidneys using the Friedman test.

Perfusate samples were analyzed for generic and kidney specific injury markers (Figure 4) as well as for the presence of pro- and anti-inflammatory markers (Figure 5).

Generic injury markers aspartate aminotransferase (AST) and lactate dehydrogenase (LDH) steadily increased over time (Figure 4). Significant differences in enzyme release were noticed with KTX 3 displaying the highest absolute enzyme levels (*p* < 0.001). Chemokine CXCL10 (IP-10, *p* < 0.001), trefoil factor 3 (TFF3, *p* = 0.010) that is found upregulated in chronic kidney disease (CKD) and the pro-inflammatory cytokine granulocyte macrophage colony-stimulating factor (GM-CSF, *p* < 0.001) all displayed highest levels in KTX 3 and 5 (Figure 4). Neutrophil gelatinase-associated lipocalin (NGAL) is released upon tubular injury into the perfusate. Despite a numerical higher level in KTX 3, similar levels between the five perfused kidneys (Figure 4, *p* = 0.080) were seen. Similarly, a comparable level of Cystatin C was measured in all groups. Clusterin, a protein released from multiple cell types upon injury with cytoprotective properties, was found in abundance in all groups and displayed a similar pattern over time in all kidney grafts. Osteopontin (OPN), a widely expressed protein during inflammation and arteriosclerosis, was found to be, unlike the other injury markers, highest in KTX 1 and KTX 2.

Highest levels of proinflammatory markers TNFa, IFNy, IL-1b, IL-2, IL-4 IL-5, IL-6 and IL-12p70 were seen in KTX 3 and KTX 4, respectively (Figure 5). In the histological work-up, KTX 4 was found to suffer from fungal contamination. A steep increase of these cytokines especially towards the end of the 48 h perfusion duration might reflect the presence of fungal contamination (Figure 5). Anti-inflammatory IL-10 expression spiked after 6 and 20 h in DBD and DCD organs, respectively. In addition, IL-10 levels increased after fungal contamination in KTX 4.

### Macroscopical and Histological Assessment

**FIGURE 6:**
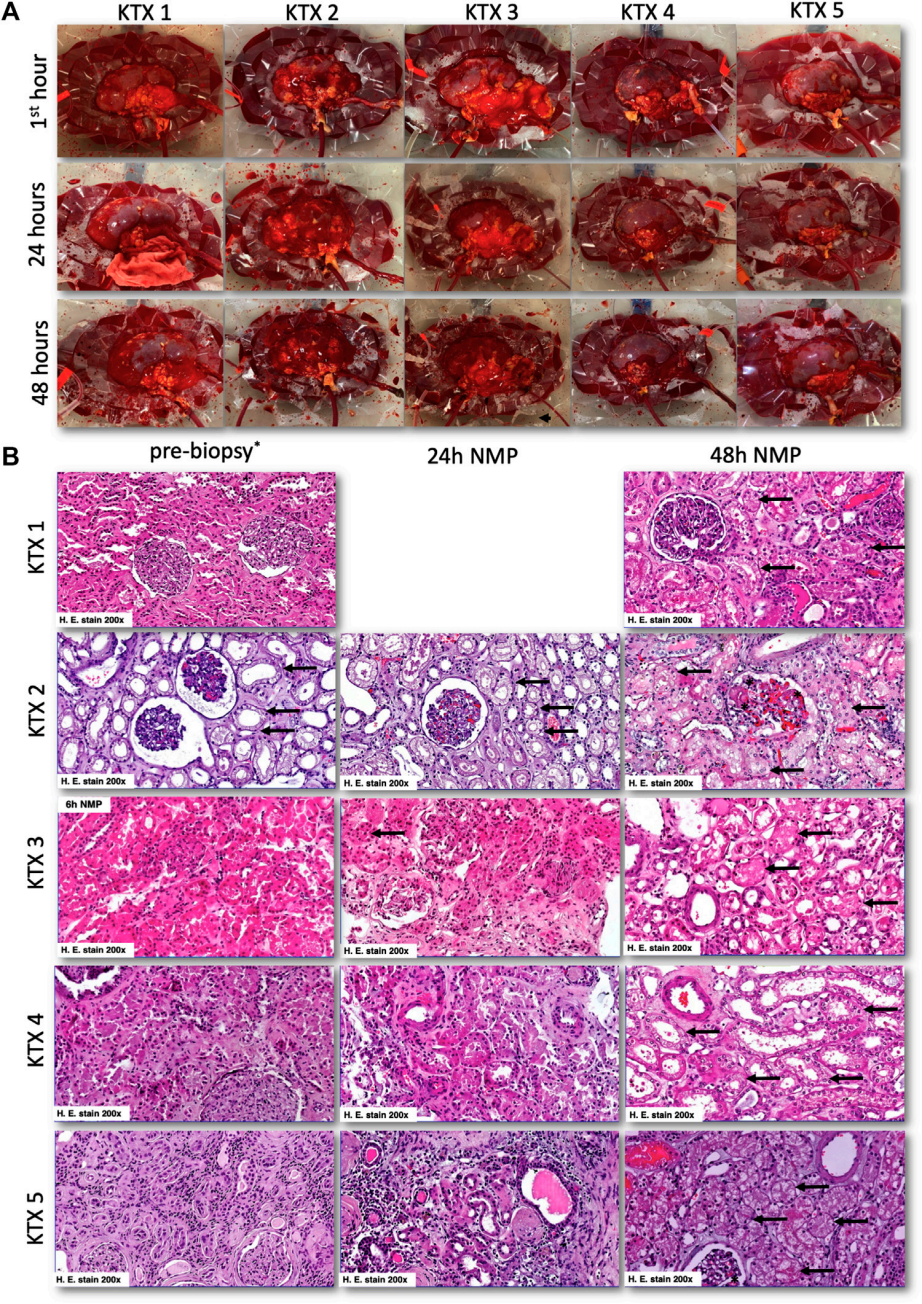
Macroscopical and histological appearance of normothermically perfused kidney grafts. **(A)** Macroscopic aspects of normothermically perfused kidneys. The upper row shows a representative image in the first hour after reperfusion, the middle row after 24 h and the lower row after reaching the endpoint at 48 h. **(B)** Representative histologic images of discarded human kidney grafts pre-NMP (left row), after 24 h of perfusion (middle row), and at 48 h of perfusion (right row). In the pre-biopsy, acute tubular damage (black arrows) with glomerular collapse was seen. This tubular damage then slightly progressed in the first 24 h of NMP (black arrows). After 48 h of NMP, again a slightly progressive tubular damage was seen together with glomerular endothelial swelling. At 48 h, 2% of glomeruli displayed signs of thrombotic microangiopathy (black asterix). No 24 h biopsy was taken in KTX1, and no pre-biopsy was available for KTX 3. For the latter one, we showed histological damage after 6 h of NMP to demonstrate progressive tubular damage. H, hours; NMP, normothermic machine perfusion.

All kidneys had a good reperfusion. Only the two poorly perfused kidneys, KTX 3 and KTX 4, showed some purple cortical areas (Figure 6). These vanished over the first 6 h of perfusion and further homogenous reperfusion was achieved until the end of the 48 h experiment. Despite favorable perfusion dynamics, macroscopic appearance of KTX 2 was the worst in our series, and KTX 4, despite a favorable appearance, was overall performing poorly.

All kidneys displayed unspecific acute tubular injury that showed moderate progression over the course of the 48 h of perfusion. No glomerular and/or tubular necrosis. In biopsies of kidneys perfused for 48 h, a small fraction (2%) of glomeruli had signs of acute thrombotic microangiopathy (Figure 6B). No evidence of thrombotic events were found at earlier timepoints. Focal oidia of the *Candida* type were present in tubules and glomeruli of KTX 3.

## Discussion

This study explored normothermic machine perfusion of human discarded kidney grafts using the Kidney Assist device and a blood based perfusate for a duration of 48 h. Perfused kidney grafts varied substantially in donor characteristics. While KTX 1 would have been transplantable without presence of malignancy in the contralateral kidney, the other kidneys were considered too marginal to be transplanted. Preexisting kidney injury, as reflected by the Remuzzi score, did not correlate with behavior during NMP.

Irrespective of donor characteristics, reason for discard, ischemic time and cold storage method, stable normothermic *ex vivo* perfusions were achieved for the whole 48 h perfusion duration. Similarly, as demonstrated earlier with 24 h perfusion experiments [7, 9], urine recirculation led to a stable perfusate composition over the prolonged perfusion time and subsequently no perfusion had to be terminated early. In two kidneys the targeted MAP of 90 mmHg resulted in such a high flow that it had to be lowered to 80 mmHg in order to stay within the technical limits of the used perfusion device. Unlike in other studies [10], flow rates did not correlate with other surrogate markers of function like lactate clearance and levels of injury markers in our series. That perfusion parameters are not necessarily reflecting clinical outcome was also reported by Hosgood et al. [2] The group perfused the kidneys of an uncontrolled DCD donor for 1 h normothermically before transplantation. Despite stable perfusion, favorable macroscopic appearance and urine output both organs experienced primary non function. As a result, the authors questioned whether their standard perfusion duration of 1 h might be too short to properly perform a pre-transplant assessment. In our series, both kidneys with poor perfusion as contributing factor for discard demonstrated the least favorable perfusion characteristics and prolonged perfusion did not alter dynamics. In addition to pre-existing thrombi, that are residual from retrieval, DeRito et al. [11] described the formation of cold storage-induced microvascular obstructions that are building up with prolonged cold storage and that might also negatively impact perfusion. They could demonstrate that the addition of plasminogen and rt-PA was able to successfully lyse these plugs. Eventually this treatment led to a significant reduction in renal injury markers, lower intrarenal resistance as well as higher urine output. In our experiment, enoxaparin, a low-molecular-weight heparin, was used as anticoagulant. A single application led to stable anti-FXa levels throughout the perfusion. Despite being eliminated renally, a clear decrease of anti-FXa activity was only observed in KTX 1.

The two poorly perfused organs, KTX 3 and KTX 4, demonstrated the highest need for exogenous glucose supplementation and accumulation of lactate, while better performing grafts, like KTX 1, 2 and 5 displayed stable or decreasing perfusate lactate levels and lower glucose supplementation needs indicating that higher glucose consumption might not necessarily indicate better metabolic activity. Similar to our findings *ex vivo*, lactate clearance has previously been shown to correlate with posttransplant renal function [12].

Urine output was present in all of our kidney grafts irrespective of donor type, CIT, cause of death and reason for discard. As already reported by others [12], the presence of urine did not seem to be an indicator for kidney graft quality. In addition, all kidneys were reabsorbing sodium, at least for the first 24 h of perfusion and most even beyond this point challenging the value of tubular sodium reabsorption as quality discriminator. Three out of five kidneys had comparable potassium urine and perfusate levels 24 h after perfusion. Only KTX 2 and KTX 5 showed significant urine potassium excretion beyond this point and strikingly KTX 1, a supposedly good quality kidney, showed poor potassium excretion.

By measuring different injury markers in the perfusate during the perfusion, we could detect some interesting dynamics. Firstly, NGAL which is a commonly used kidney injury marker [13], did not show significant differences in levels in our series. More generic markers like AST and LDH have been described to correlate with outcome after transplantation [12, 13] and were exceptionally elevated in our DCD organ. CXCL10, TFF3 and GM-CSF as well as proinflammatory markers TNFa, IFNy, IL-1b, IL-2, IL-4 IL-5, IL-6 and IL-12p70 were elevated in KTX 3 and 4. While KTX 3 had elevated perfusate levels already early, most markers increased in KTX 4 at much later timepoints. This differences in dynamics might reflect on the different reasons for poor organ quality. Finally, histologic assessment revealed, besides acute tubular injury, no glomerular and/or tubular necrosis. Thrombotic events were found in a small fraction of glomeruli after 48 h of NMP, but not at earlier timepoints. The progressive increase of tubular damage together with the presence of glomerular thrombotic events after prolonged perfusion for 48 h might indicate time limits for *ex vivo* perfusion and/or are potential signs of perfusate exhaustion.

Kidney NMP has safely been translated in the clinical setting by various groups. All applications, however, are thus far limited to short (1–3 h) perfusion durations [1, 3, 5, 6, 10, 14–16]. Novel insights from a magnetic resonance imaging study found, that perfusion during NMP takes at least 1–2 h to reach the renal cortex in a range comparable to *in vivo* and authors warned from over-interpretation of quality assessment markers for NMP at early timepoint as they may not reflect actual physiology [17]. This phenomenon might also be reflected in the dynamics of biochemical markers in the present study, where similarly low levels were found in early sampling points and relevant differences were only apparent after longer perfusion durations.

Limitations of this study include the low number of kidneys that have been perfused together with the huge variety in organ quality and donor characteristics. In addition, none of these kidneys has been transplanted and thus, no correlation to a clinical outcome can be made. Despite using the same standard recipe for all kidney perfusions, perfusate composition varied between different runs. Contributing factors might include differences in RBCs (volume, age, potassium and lactate content), tubular reabsorption capacity, urine production and sampling, and the need for exogenous buffering (depending on initial lactate and clearance). As perfusate compositions and perfusion device in different centers vary, comparison of these data to others might be limited [18].

**FIGURE 7:**
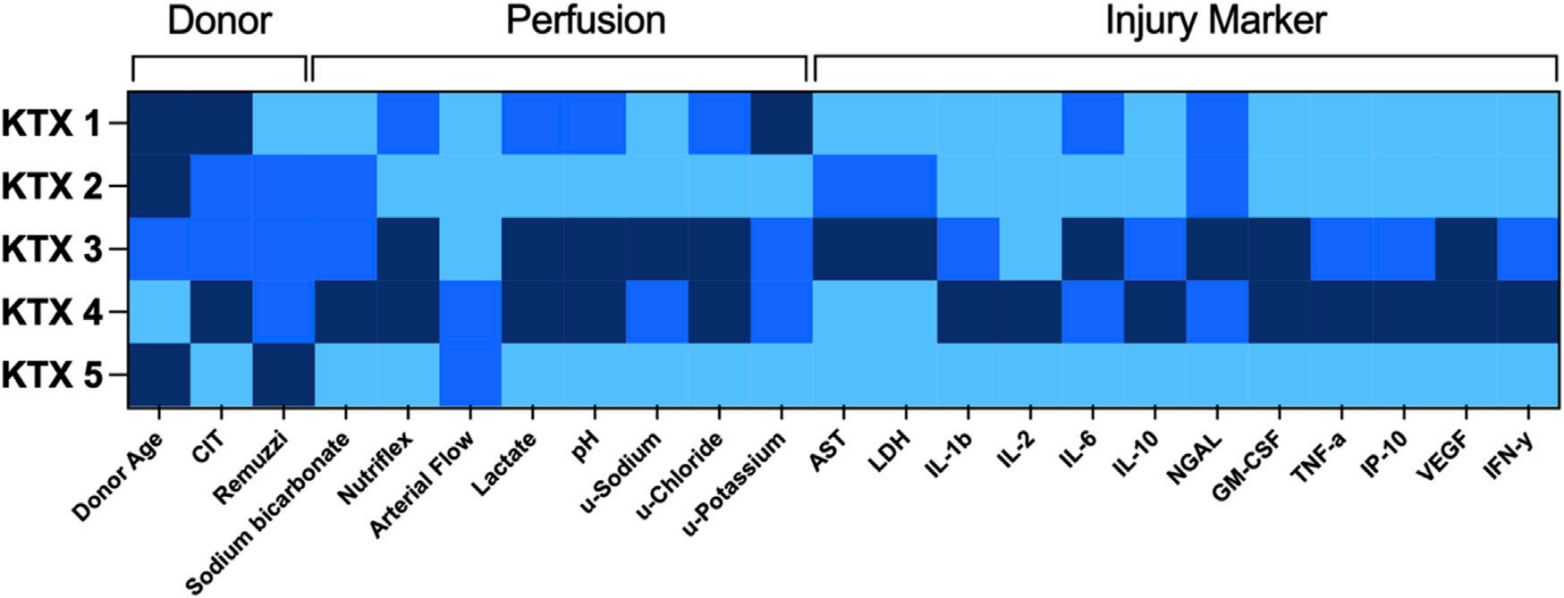
Heat map of donor characteristics, perfusion parameters and injury markers. Single factors were graded according to a semiquantitative three-tier scale (light blue = good/short/low, medium blue = intermediate, dark blue = bad/long/high). AST, aspartate aminotransferase; CIT, cold ischemic time; u, urine; GM-CSF, granulocyte macrophage colony-stimulating factor; IFN-y, interferon gamma; IL, interleukin; IP-10, CXCL10; LDH, lactate dehydrogenase; NGAL, Neutrophil Gelatinase-associated Lipocalin; TNF-a, tumor necrosis factor alpha; VEGF, vascular endothelial growth factor.

In this series of human kidney perfusion, lactate dynamics, pH, potassium excretion, as well as upregulation of injury markers show a comparable dynamic over the 48 h perfusion. By using a heat-map for donor and perfusion characteristics as well as injury markers (Figure 7), it is apparent that KTX 3 and KTX 4 are consistently performing poorer than the other three kidneys. Next in line regarding overall performance comes KTX 1, followed by KTX 2. KTX 5 had the most beneficial perfusion and injury profile.

The two kidneys with consistently undesirable profiles had the most extended donor profiles with KTX 3 being from a DCD organ with poor perfusion after retrieval, and KTX 4 from a DBD organ with poor perfusion from a septic donor on RRT and vv-ECMO. Despite lacking correlation to clinical outcome, the incorporated parameters are, as previously described by others, possible quality indicators for kidney on NMP. Prolonged perfusion might help to better identify perfusate dynamics. Long-term, longer than 24 h, *ex vivo* perfusion of the kidney, however, might be limited by accumulating tubular damage as well as *de-novo* glomerular thrombotic events when currently available devices (clinically licensed for 6 h kidney NMP only) are applied. Optimization of the perfusate might be key to improve kidney NMP outcomes further.

## Data Availability

The original contributions presented in the study are included in the article/supplementary material, further inquiries can be directed to the corresponding author.

## Abbreviations

AST, aspartate aminotransferase; CKD, chronic kidney disease; DCD, cardiocirculatory death donors; ECD, extended criteria donors; GM-CSF, granulocyte macrophage colony-stimulating factor; HMP, hypothermic machine perfusion; HTK, histidine-tryptophan-ketoglutarate; LDH, lactate dehydrogenase; NGAL, neutrophil gelatinase-associated lipocalin; NMP, Normothermic machine perfusion; TTF-3, trefoil factor 3; vv-ECMO, veno-venous extracorporeal membrane oxygenation.
